# An Automotive Ferrofluidic Electromagnetic System for Energy Harvesting and Adaptive Damping

**DOI:** 10.3390/s22031195

**Published:** 2022-02-04

**Authors:** Tadas Lenkutis, Darius Viržonis, Aurimas Čerškus, Andrius Dzedzickis, Nikolaj Šešok, Vytautas Bučinskas

**Affiliations:** Department of Mechatronics, Robotics and Digital Manufacturing, Faculty of Mechanics, Vilnius Gediminas Technical University, 10223 Vilnius, Lithuania; tadas.lenkutis@vilniustech.lt (T.L.); darius.virzonis@vilniustech.lt (D.V.); aurimas.cerskus@vilniustech.lt (A.Č.); andrius.dzedzickis@vilniustech.lt (A.D.); nikolaj.sesok@vilniustech.lt (N.Š.)

**Keywords:** semi-active damping, energy harvesting, smart liquid

## Abstract

Vibration energy harvesting is receiving significant interest due to the possibility of using extra power in various machines and constructions. This paper presents an energy-harvesting system that has a structure similar to that of a linear generator but uses permanent magnets and magnetorheological fluid insets. The application of a standard vehicle example with low frequencies and amplitudes of the excitations was used for the optimization and experimental runs. The optimization for low excitation amplitudes shows that the best magnetic field change along the slider is obtained using differentially orientated radial magnets of 5 mm in width. This configuration was used for the experimental research, resulting in 1.2–3.28 W of power generated in the coils. The power conditioning system in the experimental research was replaced by loading resistors. Nevertheless, the initial idea of energy harvesting and a damping effect was confirmed by the circuit voltage output.

## 1. Introduction

**Figure 1:**
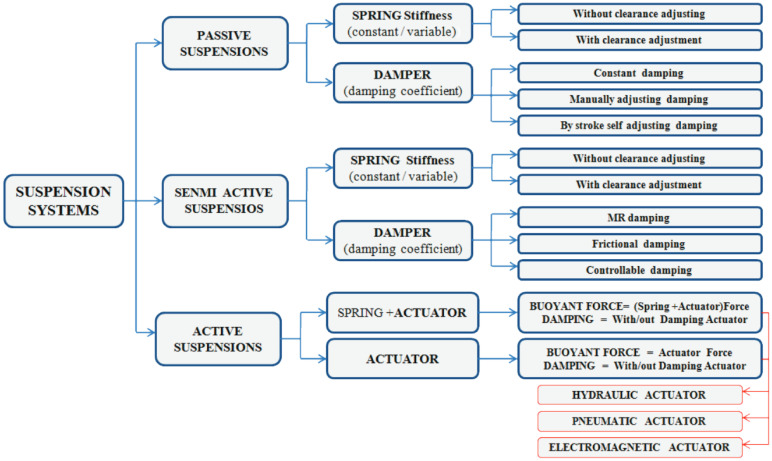
Classification of automotive suspension systems. Adapted from [9].

Mechanical vibrations are the transmission of oscillating physical energy in a solid material that rises during the operation of machines. In real-life applications, there are useful vibrations [1], where the vibrations are used to run processes, but most mechanical vibrations are harmful and destructive [2]. Vibrations should be repealed or damped to keep systems in a working condition or to achieve a higher comfort level [3]. The damping process characterized by the resistance, opposite to the direction of the system’s velocity vector, and has various forms [4]. These various types of resisting force act as damping in the dynamic process and can be classified into viscous damping [5], dry friction damping [6], material or solid or hysteretic damping [7], and magnetic damping [8]. Many applications are used to achieve the most exaggerated way of creating a damping force in different types and operation modes, but hydraulic dampers are the most common [9]. This is because of their suitability for working with higher amplitudes and compact geometric shapes [10]. Hydraulic dampers transform vibration energy to heat and dissipate it out to the surroundings. Dissipated energy, in general, cannot be re-used [11]. Hydraulic dampers are most commonly used in the suspension systems of vehicles. They increase the comfort of the ride and improve the dynamic performance of road vehicles, which is critical in certain conditions [12]. There are three types of automotive suspensions [9,13], known as passive (no damping control), active (using external power to create a damping force), and semi-active (control of the damping coefficient) (Figure 1).

Passive vehicle suspension is the simplest type of suspension because it does not actively control the damping force. Additionally, passive suspension is the most popular suspension type because of its low failure rate and low price [14]. Semi-active suspensions use external power to control the damping parameters with an electric controller. The typical semi-active suspension consists of a spring and a variable damping coefficient damper (Figure 1), where external energy is used to change the damping coefficient during the operation [15]. The active suspension uses an external power to drive the actuator in the opposite direction of the vibration force [16]. This type of suspension has a high vibration damping ratio. However, it requires lots of external energy, and controlling the hydraulic, pneumatic, or electromagnetic actuator is complex [17].

The average passenger vehicle with four wheels instantly dissipates about 600 W of damped vibration energy [18]. The power needs of semi-active suspension are about 50 W, and a correspondingly active suspension consumes 1–7 kW of external energy. However, the active suspension’s efficiency is only slightly improved compared to the semi-active version [19]. Magnetorheological fluid (MRF), which was recently introduced in semi-active dampers [20], can regulate the damping coefficient during the stroke of the damper piston by interacting with magnetic fields in real time [21]. This advantage is currently only available at the price of using external power to provide the electromagnetic coils with the required electrical current [22,23]. Damping power in semi-active suspension uses from 5% to 30% of the total damping power to control the process [24,25]. The semi-active damping process creates the possibility of collecting energy from damping rather than simply dissipating it. The amount of harvested energy can reach up to 10% of the vehicle’s driving energy [26]. While efficient damping and energy in the passive suspension are the key for best performance, in the case of magnetic damping, vibration energy can be re-used with significant value [27,28]. Furthermore, the semi-active suspension defines its relative position or velocity within the stroke during driving, and it is possible to use these data for damping control [19,29]. Usually, these systems that are mounted outside of the damper body add extra elements to the vehicle design. Therefore, a novel concept with an one-in-all damper and sensor has the space for the actual design.

Vibration energy can be harvested by a linear, permanent magnet electromagnetic generator [30]. It consists of a linear slider with permanent magnets and a series of coils in the stator, which is built over the standard shock absorber or in its place. Such installation allows the harvesting of the energy for general needs, but, at the same time, the harvested energy can be used as damping power. Most of the linear generators have an efficiency of about 40–50% [31]. The time pattern of the voltage on the linear generator coils reflects the vehicle damper movement; therefore, it can be used as a feedback signal for closed-loop control, and controlling the electrical load of the coils can be a way to control the damping coefficient.

In our research, we aimed to find a solution for vibration energy harvesting and partial damping. Part of the harvested energy was re-used to generate the damping force. The control of a damping force was made by using regulated shunting resistance. This paper describes the concept of a semi-active damper, the optimization of the magnetic circuit in the slider, and experimental testing of the prototype damper.

## 2. The Concept

The general idea of this research was to create a system and methodology for adaptive damping in a dynamic system from harmful vibrations, to utilize the harvested vibration energy to control damping, and deliver excess power externally through the energy conditioning system. Electrical damping is inefficient in the range of low-frequency vibrations due to the low speed of the electromagnetic field change. Therefore, the vehicle suspension requires some extra damping arrangements. This paper covers an exceptional electrical damping process; issues of power electronics are outside of this article’s scope.

Vehicles obtain kinematical excitation of the entire body from road inequalities through the suspension system, which reduces vibration amplitudes and resulting acceleration to the vehicle’s mass center. The amount of vibration energy necessary to dissipate in a suspension is significant; therefore, harvesting this energy is a means of energy conservation and damping efficiency improvement.

A damper (shock absorber) in the vehicle is typically employed using linear movement, where the oscillations act along its axis. The automotive vibrations are specified by a low frequency (0–20 Hz), a comparatively low amplitude (0–25 mm) [32], and random excitation. The main obstacle in using a linear generator in the vehicle’s suspension is the variable linear speed of the slider during the vibration period. When the linear speed becomes lower at the ends of the stroke, the generator stops generating power. Therefore, the existing solutions have a low-power output [33]. A possible solution by which to improve energy harvesting efficiency in the case of low-speed electric machines with permanent magnets is decreasing the pole period [34].

Direct implementation of the permanent magnet electric machine in the damper causes many technical problems in the design. Such design requires lubrication, and lubricating oil should not disturb the magnetic chain of the electric machine. On the other hand, the electric-created resistance force is not permanent and disappears at a low speed; therefore, there is a need for an extra damping system to create a damping force. Thus, a regular hydraulic damping force is applied as a base damping force, and magnetic damping from the energy generation system is an auxiliary damping force that is easily controllable. Its size has a linear dependence on the vibration amplitudes and speed. Accordingly, the magnetic damping force appears with a higher velocity within the damping system.

The implementation of MRF allows us to solve several problems. It allows us to decrease the pole period because liquid takes the shape of the internal arrangement inside the shock absorber body contrary to fragile solid ferromagnetic materials. The shock absorber with MRF utilizes this smart fluid as magnetic media. The smart fluid’s magnetic behavior is ensured due to the nanoparticles with ferromagnetic properties dispersed in the synthetic hydraulic oil.

Smart fluid excels in three important characteristics: (i) magnetic field transfer, (ii) lubrication, and (iii) the ability to change viscosity. Additionally, due to the presence of the ferromagnetic nanoparticles, which can be magnetically polarized, MRF operates as a bulk ferromagnetic material with regulated polarization. Hence, MRF became useful to change the oil’s viscosity and, while polarized, to induce an electric current in a conductor in a similar way to that of the polarized bulk ferromagnetic material.

Our concept takes the linear permanent magnet actuator as an inspiring prototype [35], from which we designed the system of a stator and slider. The main difference between the current and prototype solutions is that we replaced some of the permanent magnets of the slider with MRF containers. These are open to the outside diameter of the slider so the MRF can contact the inner wall of the stator. These open spots of the slider develop a regulated friction coefficient with the stator, which changes as the net magnetic field changes. The set of electromagnetic coils is dedicated for both harvesting the induced electromotive energy and the modulation of the magnetic field acting over the MRF. The presence of the permanent magnets is required to maintain the permanent polarization of the MRF.

**Figure 2:**
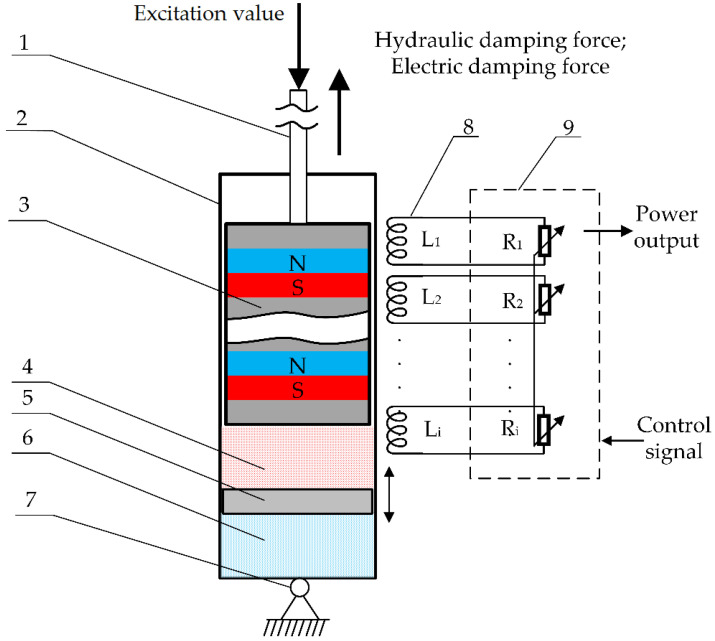
Conceptual prototype structure. 1—slider rod; 2—housing; 3—slider; 4—magnetorheological fluid; 5—separating piston; 6—air chamber; 7—flexible fixture; 8—coil set; 9—power conditioning system.

The harvester concept model (Figure 2) consists of three parts: a slider with a magnetic circuit containing MRF and permanent magnets, a hydraulic chamber to ensure the main damping force, and an air chamber to balance the space shift of the slider’s movement. The electromagnetic damping force prevails at a higher velocity of the shock absorber, and the hydraulic damping dominates at a lower velocity. The balance between the hydraulic damping force and the magnetic damping force changes as the velocity of the vibrations changes.

The experimental damper–harvester has fixed and moving parts. The moving part consists of a slider rod (1) connected to the applied excitation on one side and the slider with magnets inside connected to the other side (3) to create a permanent magnetic field. The harvester, housing (2) through fixing point (7), is fixed to the frame. The coil windings (8), consisting of eight pieces, transmit the output power to the power conditioning system (9), which controls the harvested electric power and damping process. Inside the housing, there is placed an MRF chamber (4) for the hydraulic damping process, separated with a separation piston (5) to balance the geometrical movement of the slider with the air chamber (6).

The kinematical excitation is applied to the slider rod (1) and attached to the slider (3) vertically. As the slider with the permanent magnetic field (3) moves through the coils (8), it thus generates electric energy in the coils and transmits it to the power conditioning system (9). This kind of process replaces the damping process in which energy is dissipated as a heat; instead, the energy can be harvested and utilized.

## 3. Optimization of the Electromagnetic System

The slider’s low linear speed and vibration frequency call for the optimization of the magnetic circuit to maximize the magnetic flux variations. A sufficient magnetic field alteration velocity can be achieved by changing the slider’s magnetic structure as described in Section 3.1 and the magnets’ pole positions (Section 3.2). The magnetic strength and materials have a significant impact on the results. Thus, the magnetic pole periods and the configuration of the magnetic poles require optimization.

The optimization goal (Table 1) is to find the best construction for the sliders and coils, where the magnetic flux *dB_x_* maintains maximum variations under given conditions of motion, and the coils have the optimal dimensions and electrical parameters.

### 3.1. Optimization of the Magnetic Pitch

The effectiveness of the linear generator depends on a few functional parameters such as slider speed, slider pitch size, size of the magnetic field, winding quantity, and the diameter of the wire. The operation conditions of the damper (0–10 Hz, 0–25 mm sporadic travel) limit the choice of the slider parameters to lower distances between the magnet poles, making the pole pitch and pole placement period in the slider the key efficiency parameters. This optimization procedure defines the disposition and dimensions of the magnets in the slider.

**Figure 3:**
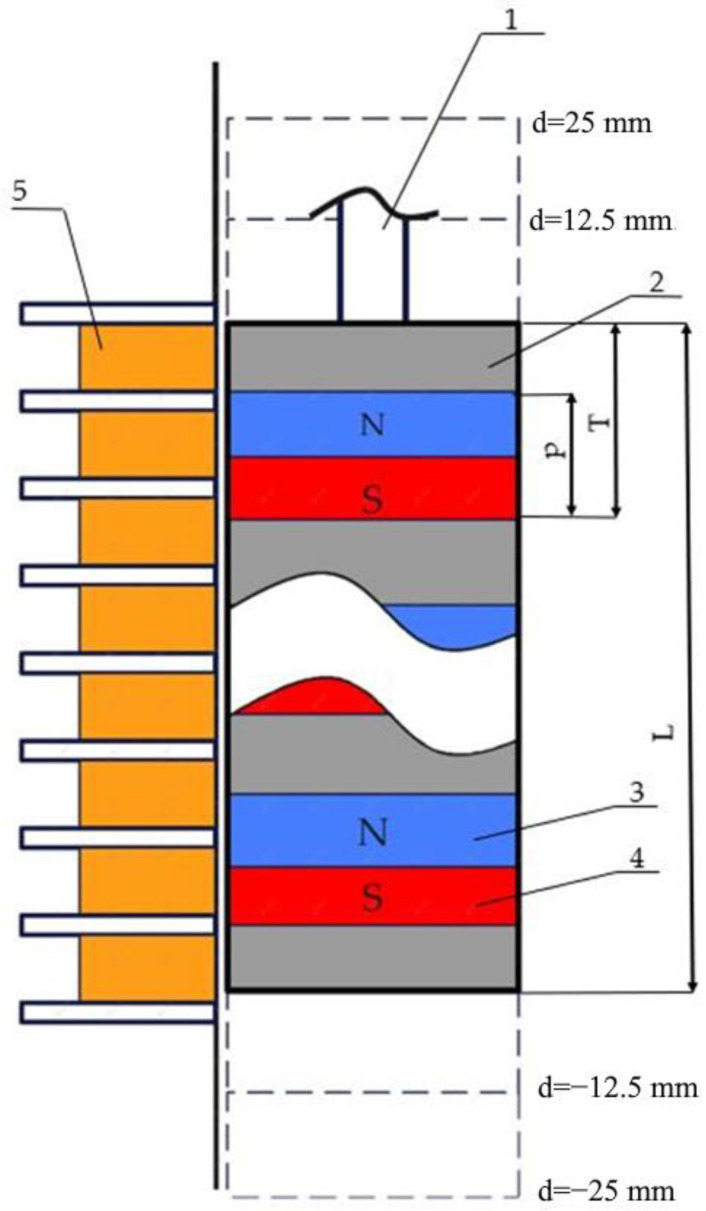
Schematic positions of permanent magnets in the slider. 1—slider rod; 2—non-magnetic material; 3—north magnetic pole; 4—south magnetic pole; 5—coils. Here, L—length of the slider, p—magnet height, and T—period of the segment (magnet plus non-magnetic insert).

The sign of optimality was the theoretically calculated amplitude and consistency of the electromotive force (EMF) time pattern. The schematic model of the slider used for the pole pitch p and pole period T optimization is provided in Figure 3.

For geometric reasoning, the electromotive force *E_mf_* equation for the single-phase linear generator can be approximated as follows:

(1) $$E_{mf}=k\cdot \sin\left(\pi \;\cdot \frac{d}{p}\right)\cdot v\left(t\right);$$

**Figure 4:**
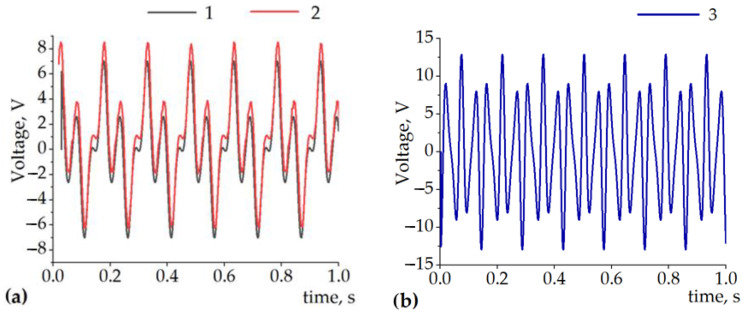
(**a**) 1—measured and 2—simulated (shifted + 1 V) output of a single coil when T = 18 mm, p = 12 mm, the amplitude of the sinusoidal excitation was 12.5 mm, and oscillating at 6.6 Hz.; (**b**) 3—simulated voltage under equivalent conditions with T = 10 mm and p = 5 mm.

Here, *ν(t)* is the velocity of an excitation value, and *k* is the voltage constant relevant to a particular design. In our case, as the design itself was uncertain at this point of the research, we used the experimentally defined *k* value for the simulation. Figure 4a shows the simulated and measured output voltage of the single-coil when the slider with T = 18 mm and when *p* = 12 mm was harmonically excited at 6.6 Hz by a 12.5 mm stroke. The circuit was loaded with 1000 Ω resistance, and the coil had 600 windings (for a more detailed explanation of the experimental setup, see Section 4 of the present paper). The output voltage graphs were shifted by 1 V for better visual perception.

One can note that the construction with the parameters T = 18 mm, p = 12 mm, and 12.5 mm excitation amplitude (Figure 4a) has a pole pitch smaller than the excitation amplitude; under this condition, less than a single period of the slider moves through the coil. Therefore, the voltage patterns are multi-frequency and non-sinusoidal. In this case, the temporal pattern of the voltage has significant regions where the local voltage value is low.

It can be observed from Figure 4b that, upon the decrease in the pole pitch to p = 5 mm and the period to T = 10 mm, it will reach the condition where the excitation amplitude (12.5 mm) is larger than the period of the slider (10 mm). Therefore, the overall peak-to-peak amplitude of the voltage will increase (due to the higher velocity of the magnetic flux variation); additionally, the overall temporal pattern of the voltage will become more consistent with the absence of regions with low voltage. A further decrease in the period and pole pitch would increase the amplitude and consistency of the voltage again. Going below 5 mm in magnet width can limit the choice of suitable and available parts and/or materials.

### 3.2. Optimization of the Magnetic Potential Distribution along the Slider with Magnetorheological Fluid Containers

Since the MRF does not possess any permanent polarization, it is essential to correctly orient and assemble the magnetic elements of the slider so that the magnetic potential is distributed favorably along the slider axis.

**Figure 5:**
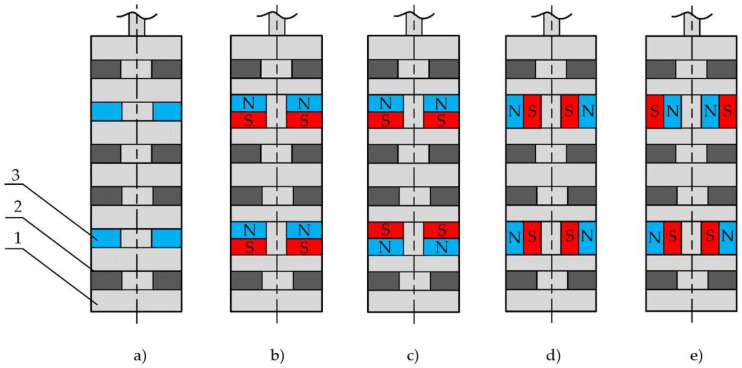
Versions of the magnet orientation in a slider (drawings are not to scale): (**a**) Overall geometry and dimensions of a slider. 1—non-magnetic material; 2—MRF insert; 3—magnet. (**b**) Unidirectional axial. (**c**) Counter-directional axial. (**d**) Unipolar radial. (**e**) Differential radial.

The overall structure of a prototype slider is shown in Figure 5a. We explored four permanent magnets’ magnetic pole orientation cases. They were unidirectional (N-S, N-S) (Figure 5b), counter directional (N-S, S-N or S-N, N-S) (Figure 5c), and two versions of the axial orientation of the magnet shown in Figure 5d,e, respectively. We used rare earth ring magnets with a 5 × 5 cross-section in all cases. The magnetic flux density inside of the magnet was 0.7 T. The goal here was to maximize the peak-to-peak amplitude of the magnetic flux density along the surface of the slider in the axial direction since the maximum magnetic flux will induce the maximum electromotive force and current in the coils.

**Figure 6:**
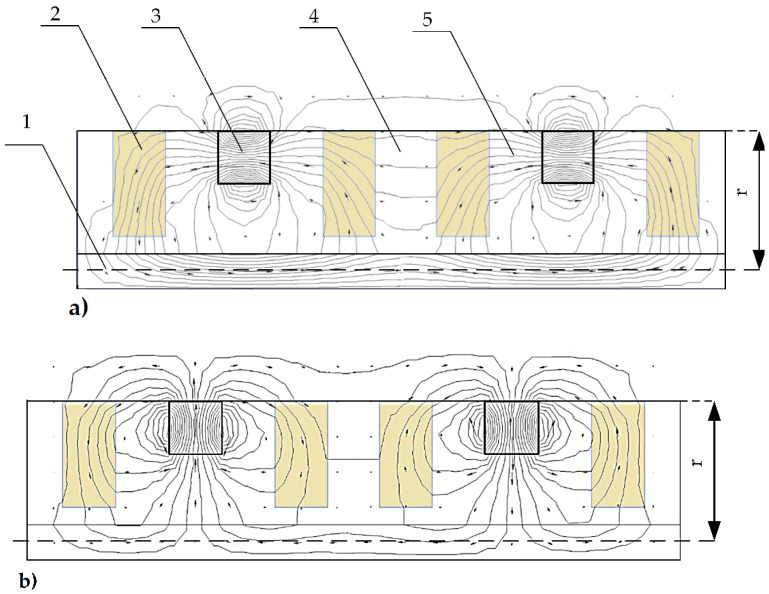
Magnetic potential lines (1 × 10^−6^ Wb interval) and magnetic flux density vectors: (**a**) Unidirectional axial orientation (see also Figure 5b). (**b**) Differential radial orientation (see also Figure 5e).

The optimization was performed by solving the magnetostatics problem using the finite element model in the software package Quickfield. A graphical representation of the magnetic potential lines and the magnetic flux density vectors in two cases, representing a unidirectional axial orientation (Figure 5b) and a differential radial orientation (Figure 5e), are shown in Figure 6a,b, respectively. The other two cases (Figure 5c,e) of magnetic field propagation had intermediate results between the shown cases and did not show any additional information; therefore, they are not shown. In Figure 5, the magnetic potential lines and magnetic flux density vectors are shown along the slider structure in the axisymmetric model (the symmetry axis is at the bottom of the structure), where 1—magnetic steel axis; 2—MRF inserts; 3—magnet; 4—non-magnetic material; 5—magnetic field lines; and r—radius of the slider. One can observe the significant difference in the magnetic field lines that go outside of the structure. The axial orientation of the magnets (Figure 6a) causes the magnetic field lines to close mostly at the inner part of the system, particularly at the damper axis (the bottom-most part of the geometry). In addition, according to the picture, the dominating direction of the magnetic flux density vectors outside of the structure point was from left to right, with very weak fractions going in the opposite direction. This indicates that only insignificant changes in the magnetic field will interact with the coils (not shown in Figure 6) during the axial motion of the slider. In contrast, the radially and differently oriented magnets (Figure 6b) provided a significant portion of the magnetic field to the outside of the structure, and the magnetic flux density vectors changed their strength and direction along the surface of the slider. We considered the last type of magnet orientation to be the optimum since the peak-to-peak amplitude of the magnetic flux density was maximum. The role of the MRF containers (2) (Figure 6) in both cases was similar to an equivalent solid ferromagnetic structure.

**Figure 7:**
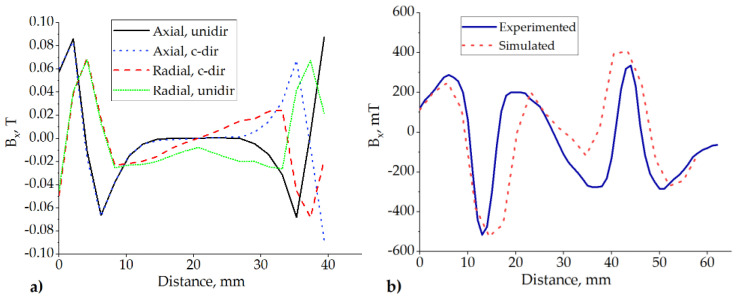
Magnetic flux density profiles along the surface of the slider: (**a**) All four magnet orientations simulated with a low resolution and reduced dimensions (see also Figure 5b–e). (**b**) Optimal setup of magnet orientations; simulation compared with the measurement.

To demonstrate the differences in the magnetic flux density amplitudes and directions, we simulated all four versions shown in Figure 5. To save computing time and effort, we simulated only the central part of the structure using coarse mesh. The results are shown in Figure 7a. It can be seen that in contrast to the other setups, the radially and counter-directionally oriented magnets (red dashed line in Figure 7a) provided the steepest change in the magnetic field density in the region of the MRF containers (between 5 and 35 mm). This effect was verified by measuring the magnetic field density of the actual prototype slider. The measurement results are provided in Figure 7b (continuous line) together with the corresponding output of the higher-resolution model (dots). The important region here is between 15 and 45 mm. Some obvious discrepancies between the measurement results and the model output can be attributed to some uncontrolled mechanical inaccuracies in the measurement setup.

## 4. Experimental Testing of the Optimized Design

**Figure 8:**
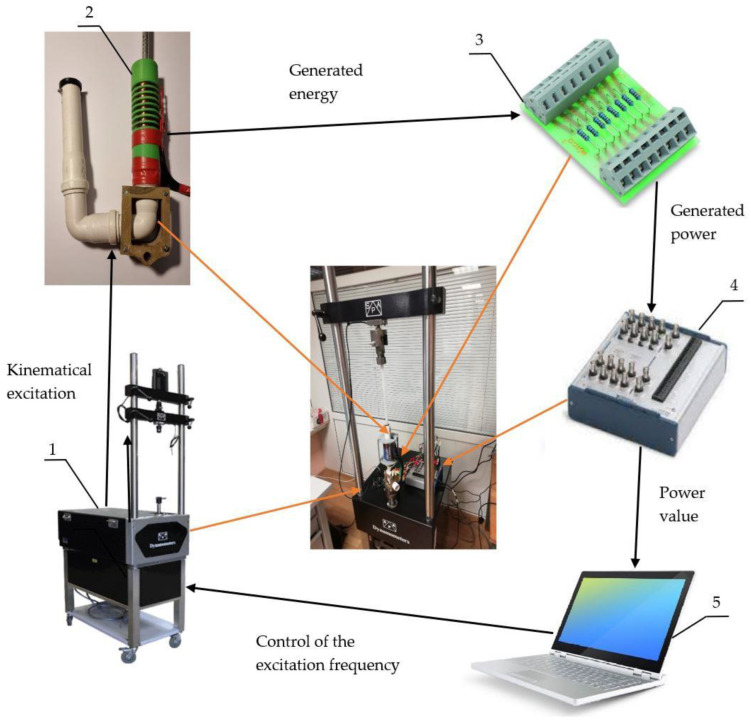
Schematic view of the experiment rack. 1—vibration source Dynamometer SPA; 2—experimental damper; 3—load resistor block; 4—data-acquiring NI-6366 converter; 5—personal computer for collecting data.

For the experimental testing, we designed a prototype. A slider with optimized dimensions (T = 10 mm; p = 5 mm) and orientation of the magnet poles (differential radial) was machined, assembled, filled with the MRF, and inserted into a plastic cylinder with a 25 mm inner diameter and eight inductive coils wound round the outside of the cylinder. The overall system’s structure is shown in Figure 8.

**Figure 9:**
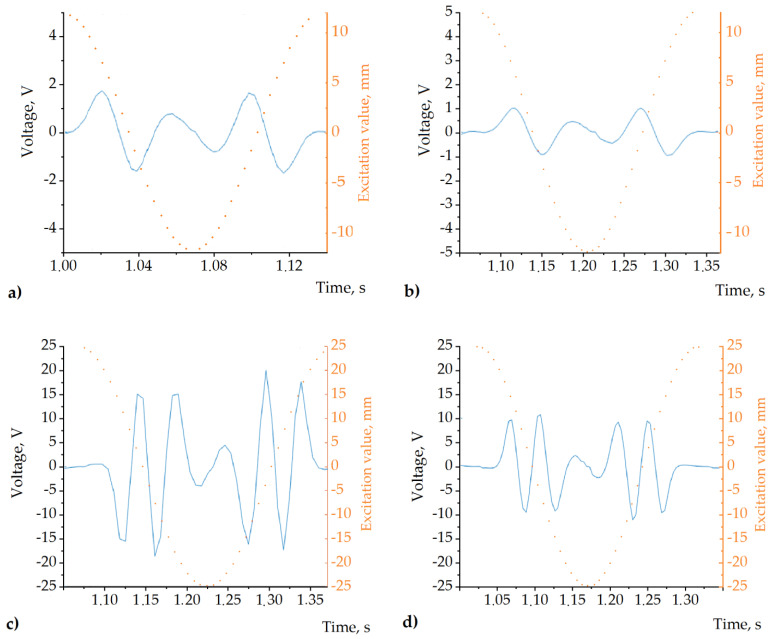
Results of the experiment. (**a**) Output voltage captured at 7 Hz and |d| = 12.5 mm. (**b**) Output voltage captured at 4 Hz and |d|= 12.5 mm. (**c**) Output voltage captured at 7 Hz and |d|= 25 mm. (**d**) Output voltage captured at 4 Hz and |d|= 25 mm excitation. The dotted line schematically illustrates the excitation value at each time moment in all cases; it corresponds to the scale annotated on the right-side axis.

Multiple coils, instead of a single coil, were installed on the prototype for research purposes. This way, it was easier to select the optimum coil position for the particular experimental setup. At the present research stage, we did not intend to use a combination of the coils for a multiple phase operation, and all the experimental results were measured from a single coil. All the coils had 600 turns of a 0.5 mm diameter copper wire. For the mechanical excitation of the experimental damper, we used the SPA Dynamometer produced by SPA Technique (Indianapolis, IN, USA). The experimental damper outputs of the coils were connected to the input of the NI6366 data acquisition board (National Instruments, Austin, TX, USA) using LabView software on a regular personal computer. The slider was harmonically excited with frequencies of 4 Hz and 7 Hz and strokes of 12.525 and 25.50 mm (corresponding to 12.5 and 25 mm sinusoidal amplitudes). The kinematic load of the suspension was taken from the average road roughness values [36]. Additionally, the useful available energy was measured by electrically loading the coil’s output with linear resistors of 10, 100, and 1000 Ω. The patterns of the voltage captured during a single excitation cycle for 1000 Ω loads are shown in Figure 9.

Since the 12.5 mm excitation cases (Figure 9a,b) involved an interaction between the coil and approximately one half of the slider structure, the average frequency of the output voltage was as twice as low as that of the 25 mm cases (Figure 9c,d). In addition, at higher excitation frequencies, the voltage amplitude is more significant because of the greater velocity. While the full excitation value swing in the case of the 25 mm excitation amplitude was 50 mm, all the elements of the magnetic system of the slider interacted with the coil during a single excitation period. The corresponding voltage patterns (see Figure 9a,b) indicated that the slider behaves like the four-pole magnetic system when only two permanent magnets are installed (Figure 5a). The additional two poles are attributable to the magnetic properties of the MRF containers, which polarize due to the permanent magnetic field and provide two additional magnetic poles. According to our undocumented observations, in the presence of an external magnetic field, the relative magnetic permeability of the MRF used in the experiments reached values comparable to those specific to the bulk ferromagnetic materials. These observations indicate the formation of a chain-like orientation of the magnetic dipoles. This interpretation of these experimental data is in good agreement with the magnetic flux density measurements and simulations carried out in the previous section (see Figure 6b). Here, one can conclude that the MRF containers in the proposed setup are beneficial not only for regulated damping but also for harvesting electrical energy.

**Figure 10:**
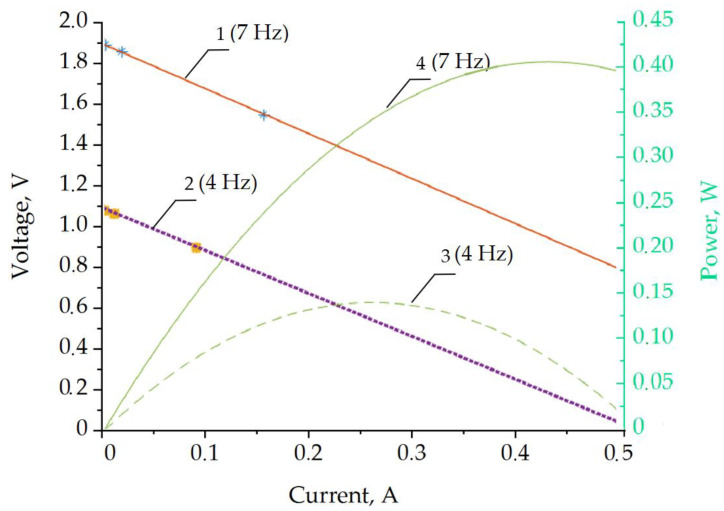
Output power characteristics of the prototype single-coil energy harvester. Load characteristics correspond to left axis: line 2–4 Hz; line 1–7 Hz. The electrical power output refers to the right (power) vertical axis: line 3–4 Hz; line 4–7 Hz.

The loading of the coil output with different resistances allowed us to estimate the useful electrical energy produced. The estimated load and power characteristics for 4 Hz and 7 Hz excitation at a 25 mm sinusoidal amplitude are shown in Figure 10. The asterisk markers indicate the measured values corresponding to the 1 kΩ, 100 Ω, and 10 Ω loads. The data in the graphs were linearly extrapolated from these experimental data points considering the root-mean-square (RMS) voltage and current values. The power output in the graph is estimated as a product of the RMS voltage and current.

The estimation of the available power indicates the optimum load of the coil, which was approximately 0.28 A in the case of 4 Hz excitation and approximately 0.43 A in the case of 7 Hz. These points also indicate the maximum useful electrical power output from the coil: approximately 0.15 W for the 4 Hz excitation per coil and slightly more than 0.4 W per coil for the 7 Hz case.

## 5. Conclusions

We designed, optimized, and demonstrated a linear permanent magnet machine with MRF elements that works with low-frequency oscillations and can be easily used for operation in semi-active automotive suspension. It has been shown that the MRF elements could work as a part of the magnetic circuit, which is used for oscillation-energy harvesting and the control of the damping coefficient. The hydraulic resistance-generated friction force should reach 80% of the damping value at 4 Hz and below, and 60% at 7 Hz, as the electrical-generated damping force increases. The values of the hydraulic resistance increase from the end of the stroke due to the increase in the smart liquid viscosity from magnetization and magnetic chain breakage.

It was found that the optimum polarization of the magnetic circuit can be achieved when the permanent magnets are oriented radially and differently for each end of the slider. The chain structure of magnetic field is broken in each stroke due to a reciprocating motion; therefore, at the end section of the stroke, the electric generation efficiency should be compensated by hydraulic damping in real cases. The optimized design of the prototype harvester enabled a significant amount of the electrical voltage to be drawn to the load, particularly up to 2 V RMS at 1000 Ω. The estimated power output of the single-coil prototype was 0.15 W during 4 Hz excitation and 0.41 W during 7 Hz excitation with 25 mm sinusoid oscillations. The overall result of the generated energy could reach up to 20–30 W for a passenger vehicle, and, for example, the theoretical calculation could be 105.2 W according to [26]. The resulting generated energy is equivalent to double the damping power [31]. In our case, this reached 40–60 W of damping power, that is, up to one-third of the maximum needed for semi-active suspension control. This theoretical and experimental research has shown that there are more possibilities for a higher power of the harvester.

The harvester structure’s main advantage is effective control, which allows the effective use of the generated power and achieves a fast response to the changes in the excitations. However, it is worth mentioning that the linear generator-type harvester has frequency zones where it does not work, so this should be taken into account when planning to control the damping using a harvested energy signal.

## Figures and Tables

**Table 1:** Formulation of the optimization task.

Title	Parameter
*Purpose function*	$dB_{x}\to max$
*Parameters*	Dimension of pitch
	Placement of magnetic field
*Limitations*	Diameter of the system
	Fixed cases of magnetic orientations

## Data Availability

The data presented in this study are available on request from the corresponding author.

## Nomenclature

**Index**	**Title**	**Unit of Measure**
MRF	Magneto rheological fluid	N/A
p	Magnet height	mm
T	Slider magnetic spatial period	mm
*ν(t)*	Speed	m/s
*k*	Voltage constant	V
R	Residence	Ω
r	Slider radius	mm
*E_mf_*	Electromotive force	V
*d*	Excitation value	mm
*B_x_*	Magnetic flux density	T
